# Association of coffee consumption and striatal volume in patients with Parkinson's disease and healthy controls

**DOI:** 10.1111/cns.14216

**Published:** 2023-04-10

**Authors:** Chao Wang, Cheng Zhou, Tao Guo, Yeerfan Jiaerken, Siyu Yang, Peiyu Huang, Xiaojun Xu, Minming Zhang

**Affiliations:** ^1^ Department of Radiology, The Second Affiliated Hospital Zhejiang University School of Medicine Hangzhou China

**Keywords:** coffee consumption, Parkinson's disease, striatum, structural MRI

## Abstract

**Background:**

Mounting studies have demonstrated that coffee consumption significantly reduces the risk of developing Parkinson's disease (PD). However, there have been few investigations about the role of chronic coffee consumption in nigrostriatal structural neurodegeneration in PD. We aimed to investigate whether chronic coffee consumption is associated with the change in striatal volume in PD.

**Methods:**

In this study, 130 de novo patients with PD and 69 healthy controls were enrolled from the Parkinson's Progression Markers Initiative cohort. Patients with PD and healthy controls were, respectively, divided into three subgroups, including current, ever, and never coffee consumers. Then, striatal volume was compared across the three subgroups. Correlation analyses were performed to assess the relationship between cups consumed per day and striatal volume. Furthermore, we included the factors that may have influenced nigrostriatal dopaminergic neurons in multiple linear regression analyses to identify significant contributing factors to striatal volume in each investigated striatal region.

**Results:**

Current coffee consumers had decreased striatal volume compared with ever consumers in controls but not patients with PD. Furthermore, the correlation analyses revealed that cups per day were negatively correlated with striatal volume in current consumers of patients with PD and controls. In addition, multiple linear regression analyses showed that current coffee consumption remained as an independent predictor of a decrease in striatal volume in controls.

**Conclusions:**

Our study showed that chronic coffee consumption was negatively correlated with striatal volume. In addition, our study showed that chronic coffee consumption was associated with the change in striatal volume in current—rather than ever coffee consumers, which suggests that the chronic effects of caffeine on striatal morphology may fade and even compensate after quitting coffee. Our study provides evidence for the effect of chronic coffee consumption on striatal volume in human brain in vivo.

## Graphical abstract

This study showed that current coffee consumption is negatively correlated with striatal volume in controls. The correlation analysis revealed that cups per day were negatively correlated with striatal volume in current consumers in both patients with PD and controls. In addition, our study firstly suggests that the chronic effects of caffeine on striatal morphology may fade and even compensate after quitting coffee consumption. Our study provides evidence for the effect of chronic coffee consumption on striatal volume in human brain in vivo.

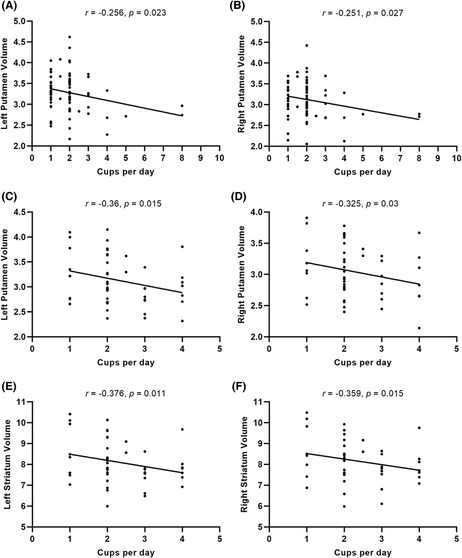

## 1 INTRODUCTION

Parkinson's disease (PD) is the second most common neurodegenerative disorder that affects 2%–3% of elderly people >65 years old worldwide.^1^ With the advent of the aging age and the extension of human life expectancy in modern society, the number of PD cases is expected to double by 2030.^2^ PD is characterized by bradykinesia, resting tremor, rigidity, and postural instability.^1^ Neuronal loss in the substantia nigra that causes a deficit of striatal dopamine and intracellular protein (α‐synuclein) accumulation that leads to the nigrostriatal pathway impairment are the neuropathological hallmarks of PD.^3^ Major contributors to PD include both genetic risk factors^4^ and environmental exposure to toxicants such as pesticides, heavy metals, and organic solvents.^5^ Nevertheless, both retrospective and prospective epidemiological studies have demonstrated coffee consumption significantly reduces the risk of developing PD.^6^, ^7^, ^8^, ^9^ Caffeine is one of the most widely consumed psychoactive substances, with an average consumption of about 200–250 mg/day/person, as a standard cup of coffee contains 100 mg of caffeine.^10^ After consumption, caffeine is rapidly absorbed through the gastrointestinal tract to the blood and then to the brain,^11^ causing alertness and reducing fatigue.^12^ The action of caffeine on the dopaminergic system is responsible for enhancing motor activity and exerting an antidyskinetic effect.^13^, ^14^ On the one hand, several studies demonstrated that chronic coffee consumption was associated with decreased functional connectivity^15^ and decreased metabolism.^16^ On the other hand, several recent studies revealed an inverse association between coffee consumption and brain volume using the data from UK Biobank.^17^, ^18^ PD is characterized by the degeneration of dopaminergic neurons in the nigrostriatal pathway.^19^ However, there have been few investigations about whether chronic coffee consumption is associated with the change in striatal volume.

The primary objective of this study was to investigate whether chronic coffee consumption could be associated with the change in striatal volume. Therefore, we systematically assessed the baseline striatal volume of current, ever, and never coffee consumers in a large sample of de novo patients with PD and healthy controls enrolled from the Parkinson's Progression Markers Initiative (PPMI) cohort. Herein, we hypothesized that coffee consumption might be correlated with decreased striatal volume.

## 2 MATERIALS AND METHODS

This study was in accordance with the approval of the Medical Ethics Committee of all PPMI sites involved and all participants were informed consent forms.

### 2.1 Participants

Data used in this study were all obtained from the PPMI database (www.ppmi‐info.org) on July 1, 2022. PPMI is an ongoing observational, international, multicenter cohort study in a large cohort. The study of the PPMI database aimed to identify clinical, imaging, genetic, and biospecimen biomarkers of PD progression. Study protocols and manuals are available on the PPMI website.

**FIGURE 1:**
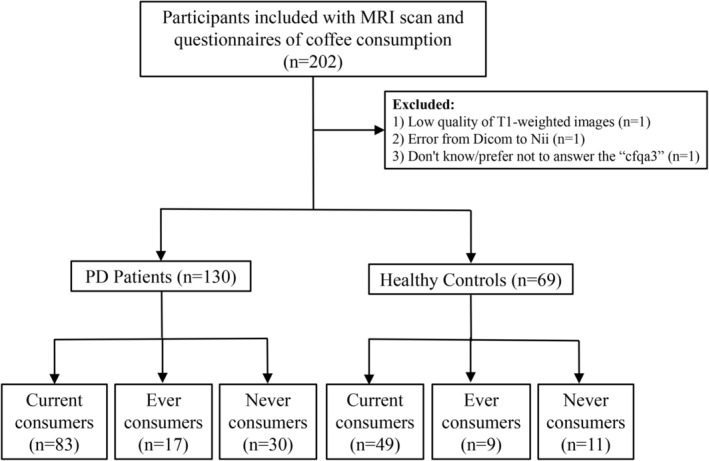
Flowchart shows participant enrollment.

In all, 202 participants with magnetic resonance imaging (MRI) scans and questionnaires about coffee consumption were enrolled in this study. The coffee consumption questionnaires included several questions ranging from “cfqa1” to “cfqa5.” “cfqa1” referred to the question “In your lifetime, have you ever regularly drunk caffeinated coffee, that is, at least once per week for 6 months or longer?” “cfqa3” referred to the question “Do you currently drink caffeinated coffee?” “cfqa5day” referred to the question “During the time you were regularly drinking caffeinated coffee, on average, about how many cups per day did you drink?” In this study, if “cfqa1 = yes” and “cfqa3 = yes,” the participants were regarded as current consumers; if “cfqa1 = yes” and “cfqa3 = no,” the participants were regarded as ever consumers; and if “cfqa1 = no” and “cfqa3 = no,” the participants were regarded as never consumers. None of the participants were current coffee consumers (cfqa3 = yes) whoever had not consumed coffee (cfqa1 = no). One participant with poor quality of T1‐weighted images, one participant with image format conversion error, and one participant who did not know/prefer not to answer the “cfqa3” were excluded. Finally, 130 de novo patients with PD and 69 healthy controls with MRI scans and the questionnaires about coffee consumption were included in this study, including 83 current consumers of PD (PD‐CC), 17 ever consumers of PD (PD‐EC), 30 never consumers of PD (PD‐NC), 49 current consumers of healthy controls (HC‐CC), 9 ever consumers of healthy controls (HC‐EC), and 11 never consumers of healthy controls (HC‐NC). The participant flowcharts of striatal volume analysis are shown in Figure 1.

### 2.2 Clinical evaluation

All participants in the PPMI cohort received the standard test battery of assessments. In addition to the demographic variables (age, gender, and education), the clinical variables were collected, including the Hoehn and Yahr (H‐Y) stages, the Movement Disorders Society Unified Parkinson's Disease Rating Scale (MDS‐UPDRS), Montreal Cognitive Assessment (MOCA), the Geriatric Depression Scale (GDS), the Scale for Outcomes for Parkinson's Disease–autonomic function (SCOPA), State and Trait Anxiety Inventory (STAI), the Questionnaire for Impulsive‐Compulsive Disorders in Parkinson's Disease (QUIP), the University of Pennsylvania Smell Identification Test (UPSIT), and the Epworth Sleepiness Scale (ESS).

### 2.3 MRI acquisition and volumetric analysis of striatum

All baseline high‐resolution 3D T1‐weighted imaging (T1WI) data were obtained at PPMI imaging centers, which were acquired according to a standardized protocol. At baseline, all PD patients were drug‐naïve. Before preprocessing, MRI images of raw DICOM format were reviewed and converted into the Neuroimaging Informatics Technology Initiative (NII) format. All NII images were preprocessed and analyzed using the CAT12 toolbox (Computational Anatomy Toolbox; http://dbm.neuro.uni‐jena.de/cat/) implemented in SPM12 (http://www.fil.ion.ucl.ac.uk/spm/software/spm12/). CAT12 served as the platform for preprocessing the structural MRI data. For processing and analysis steps, preset parameters in accordance with a standard protocol (http://www.neuro.uni‐jena.de/cat12/CAT12‐Manual.pdf) were used, applying default settings unless indicated otherwise. Then, the volume of each striatal subregion (caudate and putamen) and whole striatum were estimated according to the Neuromorphometrics and Cobra atlas, respectively. Finally, to correct for volume differences due to different head sizes, the volume of each striatal region was normalized by the total intracranial volume (TIV) of each individual, respectively: normalized striatal volume = striatal volume/individual TIV × 10^3^ (cm^3^).

### 2.4 Statistical analysis

To assess the clinical and striatal volume characteristics among subgroups in patients with PD and controls, respectively, one‐way analysis of variance (ANOVA) or t‐test was used to compare continuous variables that did follow a normal distribution, the Kruskal–Wallis test, or Mann–Whitney *U* test were used to compare continuous variables that did not follow a normal distribution, and the *χ* ^2^ test and Fisher's exact test were used for categorical variables. The Kolmogorov–Smirnov test was used to assess the normality of the continuous variables. To determine whether there was a relationship between coffee cups consumed per day and striatal imaging characteristics, partial correlation coefficients were calculated for each investigated striatal region after adjusting for age and gender in the subgroups of current and ever consumers, respectively. Furthermore, we included the factors that may have influenced nigrostriatal dopaminergic neurons in multiple linear regression analyses to identify significant contributing factors to the volume in the investigated striatal regions. In Model 1, the independent variables included age, gender, coffee consumption (current vs. ever/never consumers), and smoking history (current vs. ever/never smokers) for PD patients and controls. Moreover, Model 2 included the independent variables from Model 1, as well as age at onset, disease duration, H‐Y stages, and MDS‐UPDRS part III that were known to be associated with dopaminergic density in PD patients. Bonferroni correction was made to adjust for multiple comparisons of striatal volume characteristics. The corrected significance level is 0.05 divided by the total number of comparisons (the total number of the investigated striatal regions) provided for either patients with PD or healthy controls, that is, 0.05/6 = 0.0083 for striatal volume comparisons. Post hoc tests of one‐way ANOVA were conducted using the least significant difference (LSD) method. All statistical analyses were performed using SPSS Statistics 26 software (IBM Corporation, New York).

## 3 RESULTS

### 3.1 Demographic and clinical characteristics

In this study, a total of 130 patients with PD and 69 controls were finally included. Baseline demographic and clinical characteristics of three subgroups were summarized for patients with PD and controls in Table 1. There were no significant differences in age and gender among the three subgroups of patients with PD and controls. Education years were lower in PD‐NC than in PD‐EC (*p* = 0.006) and PD‐CC (*p* = 0.032). In addition, MDS‐UPDRS (specifically the part I, part II, part III, and total score), H‐Y stages, MOCA, GDS, SCOPA, STAI, QUIP, UPSIT, and ESS were also compared across the PD or controls subgroups, and these clinical characteristics showed no significant difference.

**TABLE 1:** Demographic and clinical characteristics of PD and healthy controls subgroups.

	PD‐CC	PD‐EC	PD‐NC	*p* Values	HC‐CC	HC‐EC	HC‐NC	*p* Values
	(*n* = 83)	(*n* = 17)	(*n* = 30)		(*n* = 49)	(*n* = 9)	(*n* = 11)	
Age (years), mean (SD)	59.9 (8.9)	63.1 (10.2)	57.4 (9.2)	0.122	60.2 (10.9)	58.7 (11.2)	60.1 (11.7)	0.937
Gender, ratio (F/M)	0.41 (24/59)	0.89 (8/9)	0.88 (14/16)	0.122	0.44 (15/34)	0.80 (4/5)	0.57 (4/7)	0.669
Education (years), mean (SD)	16.7 (2.4)	17.7 (2.8)	15.6 (2.2)	0.015	17.3 (2.5)	15.8 (2.0)	17.1 (2.8)	0.244
Coffee cups per day, median (IQR)	2.0 (1.0–2.0)	2.0 (1.0–3.0)	NA	0.776	2.0 (2.0–3.0)	3.0 (2.5–3.5)	NA	0.055
Missing (number)	3	0	NA	NA	2	0	NA	NA
Current regular smokers, *n* (%)	1 (1%)	1 (6%)	1 (3%)	NA	0 (0%)	1 (11%)	0 (0%)	NA
Age at onset (years), mean (SD)	57.9 (9.2)	61.6 (10.2)	54.7 (9.5)	0.053	NA	NA	NA	NA
Disease duration (months), mean (SD)	6.0 (5.5)	7.5 (8.5)	8.7 (8.8)	0.151	NA	NA	NA	NA
H‐Y Stages, median (IQR)	2.0 (1.0–2.0)	2.0 (1.0–2.0)	1.0 (1.0–2.0)	0.809	NA	NA	NA	NA
MDS‐UPDRS Part I, mean (SD)	5.1 (3.6)	4.9 (2.8)	4.1 (2.7)	0.332	2.9 (2.5)	3.8 (3.2)	3.0 (2.4)	0.642
MDS‐UPDRS Part II, mean (SD)	5.9 (4.2)	6.2 (3.7)	5.0 (3.9)	0.347	0.3 (0.9)	0.1 (0.3)	0.3 (0.5)	0.829
MDS‐UPDRS Part III, mean (SD)	19.5 (7.0)	22.0 (8.6)	18.8 (8.6)	0.533	0.7 (1.2)	1.2 (1.1)	0.6 (1.5)	0.518
MDS‐UPDRS Total Score, mean (SD)	30.5 (11.5)	33.1 (12.5)	27.9 (11.8)	0.366	3.9 (3.4)	5.1 (4.0)	3.9 (2.5)	0.617
MOCA, mean (SD)	27.5 (2.0)	27.2 (2.2)	27.9 (2.4)	0.45	28.0 (1.0)	28.4 (1.3)	28.6 (1.3)	0.172
GDS, median (IQR)	1.0 (1.0–3.0)	2.0 (1.0–2.5)	1.0 (0–2.25)	0.792	1.0 (0–1.5)	0 (0–1.0)	1.0 (0–2.0)	0.782
STAI, mean (SD)	62.9 (17.0)	56.1 (17.9)	61.4 (15.7)	0.323	52.6 (10.9)	61.7 (19.4)	52.6 (13.8)	0.142
QUIP, median (IQR)	0 (0–0)	0 (0–1)	0 (0–0)	0.238	0 (0–1)	0 (0–0)	0 (0–0)	0.109
UPSIT, mean (SD)	22.5 (7.9)	24.0 (7.3)	25.7 (7.5)	0.139	34.7 (4.8)	33.9 (5.9)	33.6 (4.9)	0.771
ESS, mean (SD)	5.6 (3.3)	5.7 (3.7)	5.7 (3.3)	0.994	5.4 (3.3)	5.7 (3.9)	6.3 (3.6)	0.725
SCOPA, mean (SD)	8.7 (5.3)	8.7 (4.9)	6.6 (4.7)	0.137	5.1 (2.9)	5.8 (2.5)	6.0 (3.6)	0.612

*Note*: Three PD‐CC did not know/prefer not to answer the question ‘cups per day’. Two HC‐CC did not know/prefer not to answer the question ‘cups per day’. Statistical results are reported as mean (standard deviation, SD) if the original data follows a normal distribution, otherwise as median (interquartile range, IQR).

Abbreviations: GDS, Geriatric Depression Scale; ESS, Epworth Sleepiness Scale Score; H‐Y stages, Hoehn & Yahr stages; HC‐CC, current consumers of healthy controls; HC‐EC, ever consumers of healthy controls; HC‐NC, never consumers of healthy controls; MDS‐UPDRS, Movement Disorders Society Unified Parkinson's Disease Rating Scale; MOCA, Montreal Cognitive Assessment; NA, not applicable; PD‐CC, current consumers of Parkinson's disease; PD‐EC, ever consumers of Parkinson's disease; PD‐NC, never consumers of Parkinson's disease; QUIP, Questionnaire for Impulsive‐Compulsive Disorders; SCOPA, Scale for Outcomes for Parkinson's Disease–autonomic function; STAI, State and Trait Anxiety Inventory; UPSIT, University of Pennsylvania Smell Identification Test.

### 3.2 Striatal volume characteristics

**FIGURE 2:**
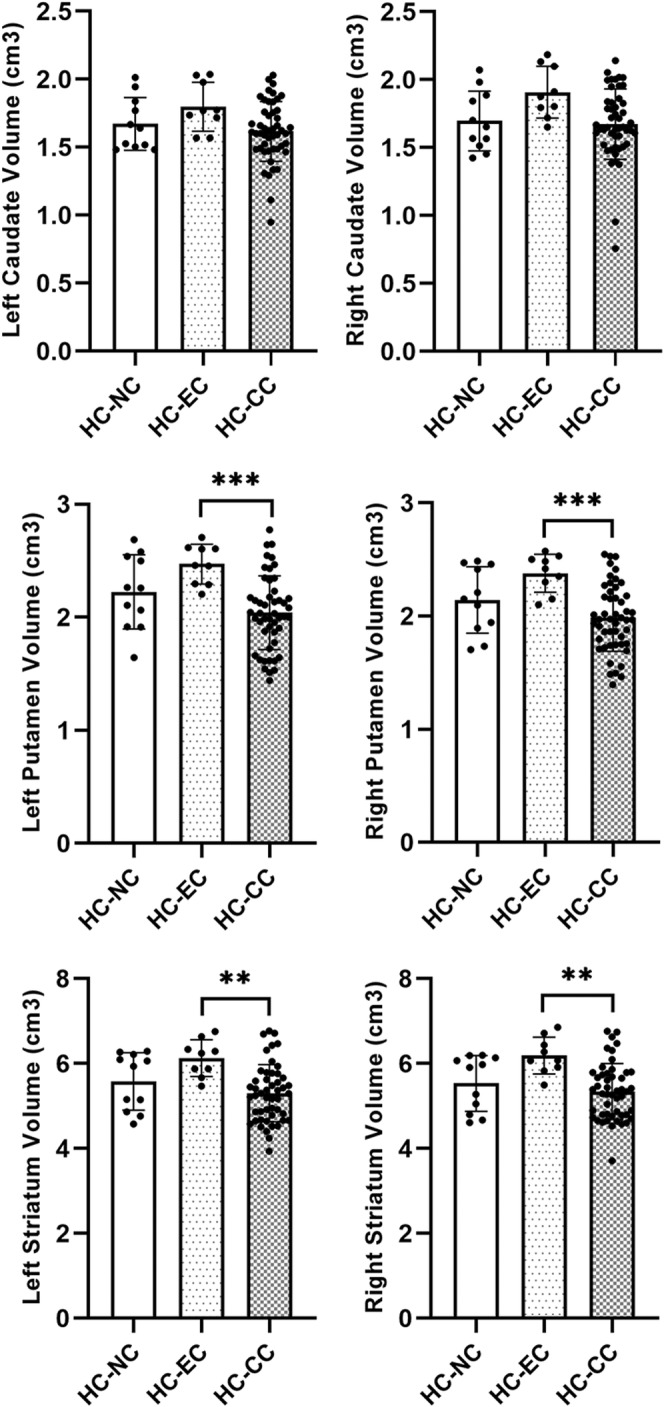
In healthy controls, current coffee consumers had a tendency of lower volume in each striatal region than ever and never consumers, including the left caudate (*p* = 0.065), right caudate (*p* = 0.038), left putamen (*p* = 0.001), right putamen (*p* = 0.001), left striatum (*p* = 0.003), and right striatum (*p* = 0.002) across three subgroups. Then, post hoc tests were further performed if *p* < 0.0083 (Bonferroni correction). Specifically, post hoc tests showed that current consumers had significantly lower volume in the left putamen (*p* < 0.001), right putamen (*p* < 0.001), left striatum (*p* = 0.001), and right striatum (*p* = 0.001) than ever consumers. **p < 0.01 and ***p < 0.001.

**FIGURE 3:**
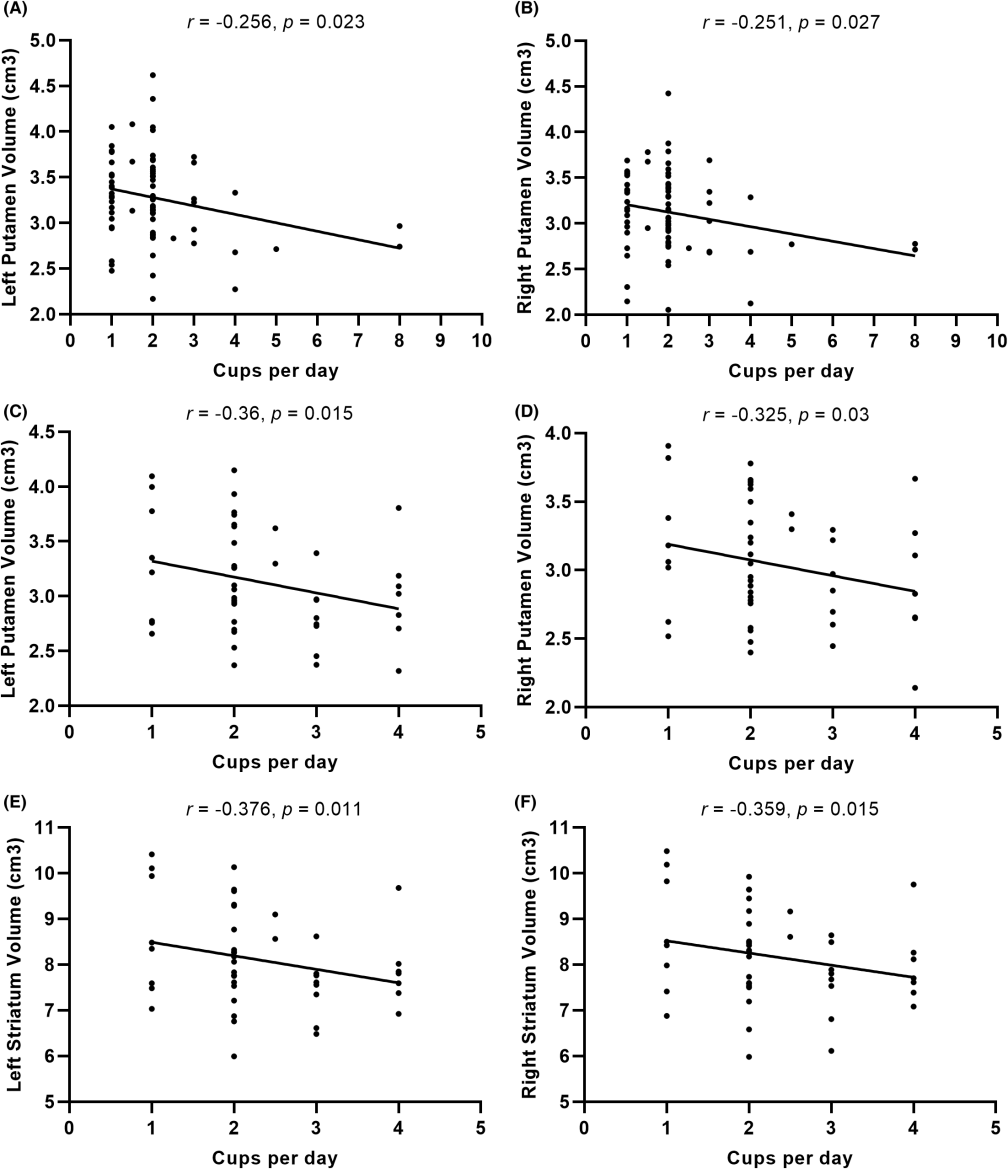
In current consumers of patients with PD (A, B), the correlation analysis showed that cups per day were negatively correlated with volume in the (A) left putamen and (B) right putamen. In current consumers of healthy controls (C–F), cups per day were negatively correlated with volume in the left putamen (C), right putamen (D), left striatum (E), and right striatum (F).

There was no significant difference of the volume in all striatal regions between patients with PD and controls (Table S1). The volume of each striatal region was further compared across three subgroups in patients with PD and controls, respectively. In patients with PD, there was no significant difference in the volume of any striatal region across the three subgroups. However, in controls, current consumers had a tendency to lower volume in each striatal region than ever/never consumers (Figure 2, Table 2), including the left putamen (*p* = 0.001), right putamen (*p* = 0.001), left striatum (*p* = 0.003), and right striatum (*p* = 0.002) across three subgroups. Specifically, post hoc tests showed that current consumers had significantly lower volume in the left putamen (*p* < 0.001), right putamen (*p* < 0.001), left striatum (*p* = 0.001), and right striatum (*p* = 0.001) than ever consumers (Figure 2). The correlation analysis revealed that cups per day were negatively correlated with volume in the left putamen (*r* = −0.256, *p* = 0.023) and right putamen (*r* = −0.251, *p* = 0.027) in PD‐CC (Figure 3A,B, Table S2). In addition, cups per day were negatively correlated with volume in the left putamen (*r* = −0.36, *p* = 0.015), right putamen (*r* = −0.325, *p* = 0.03), left striatum (*r* = −0.376, *p* = 0.011), and right striatum (*r* = −0.359, *p* = 0.015) in HC‐CC (Figure 3C–F, Table S2).

**TABLE 2:** Striatal volume between subgroups of PD patients and healthy controls, respectively.

	PD‐CC	PD‐EC	PD‐NC	*p*	HC‐CC	HC‐EC	HC‐NC	*p*
	(*n* = 83)	(*n* = 17)	(*n* = 30)		(*n* = 49)	(*n* = 9)	(*n* = 11)	
Left caudate (cm^3^)	1.67 ± 0.21	1.63 ± 0.28	1.69 ± 0.24	0.739	1.62 ± 0.22	1.79 ± 0.18	1.67 ± 0.19	0.065
Right caudate (cm^3^)	1.74 ± 0.23	1.72 ± 0.28	1.73 ± 0.34	0.974	1.67 ± 0.26	1.91 ± 0.19	1.70 ± 0.22	0.038
Left putamen (cm^3^)	2.15 ± 0.28	1.99 ± 0.39	2.11 ± 0.37	0.148	2.04 ± 0.33	2.47 ± 0.18	2.22 ± 0.33	**0.001**
Right putamen (cm^3^)	2.05 ± 0.26	1.97 ± 0.33	2.01 ± 0.31	0.571	1.98 ± 0.29	2.38 ± 0.17	2.14 ± 0.29	**0.001**
Left striatum (cm^3^)	5.48 ± 0.58	5.29 ± 0.93	5.44 ± 0.78	0.568	5.30 ± 0.67	6.13 ± 0.43	5.58 ± 0.68	**0.003**
Right striatum (cm^3^)	5.48 ± 0.60	5.39 ± 0.80	5.46 ± 0.77	0.881	5.34 ± 0.66	6.19 ± 0.43	5.53 ± 0.66	**0.002**

*Note*: Bold values indicate significant differences (*p* < 0.0083) after Bonferroni correction.

Abbreviations: HC‐CC, current consumers of healthy controls; HC‐EC, ever consumers of healthy controls; HC‐NC, never consumers of healthy controls; PD‐CC, current consumers of Parkinson's disease; PD‐EC, ever consumers of Parkinson's disease; PD‐NC, never consumers of Parkinson's disease.

### 3.3 Independent contribution of coffee consumption to striatal volume

The results of striatal volume in multiple linear regression analysis of Model 1 for PD patients and controls are presented in Table 3. In patients with PD, there was no significant association between current coffee consumption and the volume of any striatal region. However, in patients with PD, multiple regression analysis showed positive associations between gender (female) and volume in the left caudate (*ß* = 0.310, *p* = 0.001, 95% confidence interval [CI]: 0.064 to 0.227), right putamen (*ß* = 0.282, *p* = 0.002, 95% CI: 0.063 to 0.268), left striatum (*ß* = 0.286, *p* = 0.002, 95% CI: 0.158 to 0.653), and right striatum (*ß* = 0.270, *p* = 0.003, 95% CI: 0.134 to 0.613). In controls, multiple regression analysis showed inverse associations between current coffee consumption and volume in the left putamen (*ß* = −0.378, *p* = 0.001, 95% CI: −0.445 to −0.123), right putamen (*ß* = −0.367, *p* = 0.001, 95% CI: −0.396 to −0.102), left striatum (*ß* = −0.316, *p* = 0.004, 95% CI: −0.801 to −0.165), and right striatum (*ß* = −0.289, *p* = 0.008, 95% CI: −0.751 to −0.121). In addition, inverse associations were found between age and volume in the left caudate (*ß* = −0.335, *p* = 0.007, 95% CI: −0.011 to −0.002), right caudate (*ß* = −0.353, *p* = 0.005, 95% CI: −0.014 to −0.003), left putamen (*ß* = −0.364, *p* = 0.002, 95% CI: −0.019 to −0.004), right putamen (*ß* = −0.326, *p* = 0.006, 95% CI: −0.016 to −0.003), left striatum (*ß* = −0.435, *p* < 0.001, 95% CI: −0.042 to −0.014), and right striatum (*ß* = −0.429, *p* < 0.001, 95% CI: −0.041 to −0.013).

**TABLE 3:** Factors associated with volume in each striatal subregion by a multiple linear regression analysis in PD patients and healthy controls.

Striatal regions	Variables	PD (*n* = 130)			HC (*n* = 69)		
		*ß*	95% CI	*p*	*ß*	95% CI	*p*
Left caudate	Age	−0.147	−0.008 to 0.001	0.088	−0.335	−0.011 to −0.002	**0.007**
	Gender	0.310	0.064 to 0.227	**0.001**	0.037	−0.092 to 0.126	0.765
	Coffee consumption	0.078	−0.042 to 0.115	0.363	−0.203	−0.205 to 0.012	0.081
	Smoking history	−0.044	−0.319 to 0.189	0.613	0.100	−0.243 to 0.602	0.398
Right caudate	Age	−0.212	−0.011 to −0.001	0.017	−0.353	−0.014 to −0.003	**0.005**
	Gender	0.176	−0.001 to 0.195	0.052	0.041	−0.106 to 0.150	0.734
	Coffee consumption	0.044	−0.070 to 0.118	0.617	−0.181	−0.228 to 0.026	0.118
	Smoking history	−0.052	−0.395 to 0.215	0.56	0.085	−0.316 to 0.676	0.471
Left putamen	Age	−0.093	−0.009 to 0.003	0.292	−0.364	−0.019 to −0.004	**0.002**
	Gender	0.196	0.011 to 0.248	0.032	−0.001	−0.163 to 0.162	0.994
	Coffee consumption	0.170	−0.003 to 0.226	0.056	−0.378	−0.445 to −0.123	**0.001**
	Smoking history	0.005	−0.360 to 0.380	0.956	0.020	−0.570 to 0.686	0.855
Right putamen	Age	−0.146	0.010 to 0.001	0.094	−0.326	−0.016 to −0.003	**0.006**
	Gender	0.282	0.063 to 0.268	**0.002**	0.027	−0.130 to 0.166	0.811
	Coffee consumption	0.126	−0.025 to 0.173	0.143	−0.367	−0.396 to −0.102	**0.001**
	Smoking history	−0.065	−0.441 to 0.200	0.457	0.056	−0.429 to 0.718	0.616
Left striatum	Age	−0.143	−0.023 to 0.002	0.098	−0.435	−0.042 to −0.014	**<0.001**
	Gender	0.286	0.158 to 0.653	**0.002**	0.029	−0.278 to 0.364	0.788
	Coffee consumption	0.122	−0.067 to 0.411	0.157	−0.316	−0.801 to −0.165	**0.004**
	Smoking history	−0.002	−0.780 to 0.766	0.985	0.068	−0.845 to 1.637	0.526
Right striatum	Age	−0.213	−0.028 to −0.003	0.014	−0.429	−0.041 to −0.013	**<0.001**
	Gender	0.270	0.134 to 0.613	**0.003**	0.041	−0.258 to 0.378	0.707
	Coffee consumption	0.083	−0.117 to 0.346	0.329	−0.289	−0.751 to −0.121	**0.008**
	Smoking history	−0.062	−1.020 to 0.476	0.473	0.088	−0.726 to 1.732	0.416

*Note*: Bold values indicate significant differences (*p* < 0.0083) after Bonferroni correction.

Abbreviations: CI, confidence interval; HC, healthy controls; PD, Parkinson's disease; *ß*, standardized coefficient beta.

In addition, the multiple linear regression analysis of Model 2 included the independent variables from Model 1, as well as age at onset, disease duration, H‐Y stages, and MDS‐UPDRS part III for patients with PD (Table S3). In patients with PD, there was no significant association between age at onset, disease duration, H‐Y stages, MDS‐UPDRS part III, and the volume of the investigated striatal regions. However, in patients with PD, gender (female) remained independently positively associated with volume in the left caudate (*ß* = 0.325, *p* < 0.001, 95% CI: 0.069 to 0.236), right putamen (*ß* = 0.279, *p* = 0.002, 95% CI: 0.059 to 0.268), left striatum (*ß* = 0.3, *p* = 0.001, 95% CI: 0.174 to 0.677), and right striatum (*ß* = 0.269, *p* = 0.003, 95% CI: 0.129 to 0.614). Since age at onset, disease duration, H‐Y stages, and MDS‐UPDRS part III were not available in healthy controls, model 2 analysis was not performed for controls.

## 4 DISCUSSION

In the present study, we compared the striatal volume across three subgroups of coffee consumption using quantitative analyses in both patients with PD and healthy controls, respectively. This study demonstrated that current consumers had decreased striatal volume compared with ever consumers in controls. The correlation analysis revealed that cups per day were negatively correlated with striatal volume in PD‐CC and HC‐CC. Furthermore, after including the factors that may have influenced nigrostriatal dopaminergic neurons, multiple regression analyses showed current coffee consumption remained as an independent predictor of a decrease in striatal volume in controls. In addition, multiple regression analyses showed a positive association between gender (female) and striatal volume in patients with PD, and an inverse association between age and striatal volume in controls.

Dorsal striatum consists of dorsomedial striatum (caudate) and dorsolateral striatum (putamen). Caffeine is one of the most widely consumed psychostimulants.^10^ Acute exposure to psychostimulants acts to increase dopamine release in the dorsal striatum.^20^ And repeated exposure sensitizes the dorsal striatum, potentiates dopamine release, and subsequently augments habitual behavior.^21^, ^22^, ^23^ Furthermore, persistent structural alterations to the dorsal striatal dopamine system have also been reported following repeated exposure to psychostimulants that augment habits, including reduced dopamine transporter (DAT) binding, and dopamine D1 and D2 receptor binding.^24^, ^25^ Using single photon emission computed tomography (SPECT) imaging, Gigante et al. investigated the association between chronic coffee consumption and striatal DAT binding in patients with PD. However, their results showed coffee consumption was not correlated with any significant change in striatal DAT binding.^26^ Caffeine nonselectively antagonizes four kinds of adenosine receptors, i.e., A1R, A2AR, A2BR, and A3R. Of these adenosine receptors, A2AR and A1R are abundant in the striatum.^27^, ^28^ A neuroprotective effect of caffeine is well documented in experimental PD models, and is probably mediated by inhibitory A1R and facilitatory A2AR.^29^, ^30^, ^31^ Several experiments showed that caffeine pretreatment before 1‐methyl‐4‐phenyl‐1,2,3,6‐tetrahydropyridine (MPTP) administration resisted dopamine depletion in a dose‐dependent manner in mice.^32^, ^33^ In mice, the residual dopamine levels increased to 40% of control values with caffeine pretreatment (10 mg/kg 10 min), whereas it decreased to only 15% of control values without caffeine pretreatment.^33^ Furthermore, another experiment demonstrated that delayed caffeine administration could also reduce the loss of nigral dopamine cell bodies and block the nigral neurodegenerative process in rats.^34^ In the present study, we observed an inverse association between current coffee consumption and striatal volume in controls and between coffee cups consumed per day and striatal volume in current consumers of PD and controls. Recently, using the data from UK Biobank, several large‐scale studies revealed an inverse association between coffee consumption and gray matter volume.^17^, ^18^ Moreover, the finding of this inverse association was supported by a double‐blind randomized controlled trial (RCT).^35^ A total of 20 healthy male habitual caffeine consumers were recruited for the RCT study. The RCT study revealed a significant reduction in gray matter volume after 10 days of caffeine administration (3 doses of 150 mg per day) compared with 10 days of placebo. Multiple physiological mechanisms have been proposed to account for the neuroprotective effects of caffeine, such as anti‐neuroinflammation effects and antioxidant effects through blocking adenosine receptors.^36^ Caffeine facilitates synaptic transmission by antagonizing A1R, while it also attenuates long‐term synaptic potentiation by blocking A2AR,^37^ which is abundant in the striatum.^38^ The impact of caffeine on long‐term synaptic potentiation via blocking A2AR may be partially responsible for the effects of chronic coffee consumption on striatal structural change. In addition, we speculate the decreased striatal volume may also be due to dehydration, change in neuronal numbers, and change in vascular compartments caused by coffee consumption. Notably, as shown in Figure 2, the present study demonstrated that there was a significant decrease in striatal volume in current rather than ever consumers, while there was a tendency for striatal volume to increase in ever consumers. Our findings indicate that the chronic effects of caffeine on striatal morphology may also fade and even compensate after quitting coffee consumption.

In the present study, the influence of current coffee consumption on striatal volume was only detected in controls rather than patients with PD. Moreover, the correlation analysis revealed that cups per day were negatively correlated with volume in current consumers in four of the investigated striatal regions in controls but only in two of the investigated striatal regions in patients with PD. Furthermore, an inverse association was found between age and striatal volume in all of the investigated striatal regions in controls but not in patients with PD. One possible explanation for the observation that controls showed more significant results than patients with PD may be a statistical “floor” effect due to PD pathologies. In Bohnen et al.'s study, they examined associations between asymmetric hemispheric nigrostriatal dopaminergic denervation with DAT imaging and pegboard scores in patients with PD.^39^ Their results showed there was no significant correlation between pegboard scores of the most affected arm and DAT binding of the most denervated striatum, whereas there was an inverse correlation between pegboard scores of the least affected arm and DAT binding of the least denervated striatum. It was suggested that these discrepant results were caused by a statistical “floor” effect due to more severe denervation of the striatum.^39^

In addition, we observed an inverse association between age and striatal volume in controls. Consistent with our findings, a recent study revealed an age‐related decline in striatal volume in healthy individuals across the entire adult lifespan.^40^ Moreover, the present study showed a positive association between gender (female) and striatal volume in patients with PD, which suggests that male sex shows more severe atrophy and female sex confers protective benefits against neurodegeneration in PD. In fact, an early epidemiologic study has demonstrated that male sex has a two‐fold higher risk of developing PD.^41^ Structural MRI studies have evidenced sex‐based differences in PD. Male sex has pronounced cortical atrophy in several lobes,^42^ while male sex has lower subcortical volume in the thalamus,^43^ caudate, and pallidum compared with females.^44^ Possible pathophysiological mechanisms for sex differences are the sex‐related differences caused by different gene expression and sex hormones in the key players of PD pathogenesis, including the vulnerability of the dopaminergic system, neuroinflammation, and oxidative stress.^45^

In our study, we detected no significant differences of the volume in striatal regions between patients with PD and controls. At first glance, this may seem surprising, since the striatum is the central structure of dopamine deprivation in PD. In fact, our finding is consistent with previous studies,^46^, ^47^, ^48^ there were no significant differences of the volume in total striatum, caudate, and putamen between PD patients and controls. One possible explanation is that there is variability across subregions of the striatum in terms of how significantly and at what time they are dopamine depleted in PD. Khan et al.'s study showed that although PD patients had no differences in the volume of total striatum, caudate, and putamen, PD patients showed atrophy in the caudal‐motor striatal subregion compared with controls.^46^ In addition, all the PD patients included in our study were early‐stage PD. The early stage of the disease may not result in a significant enough reduction in the total striatal, caudate, and putamen volumes.

This study has several limitations. First, although several known confounding factors that significantly influence dopaminergic density were included in this analysis, it is possible that other lifestyle‐related confounding factors may have altered the results. Second, PD is characterized by the degeneration of dopaminergic neurons in the nigrostriatal pathway. However, in this study, nigral volume was not examined due to a lack of quantitative susceptibility mapping (QSM) and neuromelanin‐sensitive MRI. In future, PD patients should be scanned with QSM and neuromelanin‐sensitive MRI to investigate the association between chronic coffee consumption and nigral volume. Third, information regarding the time of the last coffee cup consumed before the imaging scan was not available. Future studies should investigate the acute effects of coffee consumption and thus explain whether acute coffee consumption influences striatal volume.

In conclusion, the present study showed that current coffee consumption was negatively correlated with striatal volume in controls but not patients with PD. The correlation analysis revealed that cups per day were negatively correlated with striatal volume in current consumers in both patients with PD and controls. In addition, our study firstly showed that there was a significant decrease in striatal volume in current rather than ever consumers, while there was a tendency for striatal volume to increase in ever consumers. Our findings indicate that the chronic effects of caffeine on striatal morphology may also fade and even compensate after quitting coffee consumption. Our study provides evidence for the effect of chronic coffee consumption on striatal volume in human brain in vivo.

## CONFLICT OF INTEREST STATEMENT

The authors have no conflict of interest to report.

## CONSENT TO PARTICIPATE

Written informed consent was obtained from all participants.

## Supporting information


Table S1.

Table S2.

Table S3.

## Data Availability

The data that support the findings of this study are available from the corresponding author upon reasonable request.
