# Supplementary material for: Association of coffee consumption and striatal volume in patients with Parkinson's disease and healthy controls
Source: CNS Neurosci Ther. 2023 Apr 10;29(10):2800–10. doi: 10.1111/cns.14216 (PMC10493673; doi:10.1111/cns.14216)
Supplement: Supplementary file 1 — Table S1. [file CNS-29-2800-s001.docx]

**Table S1. Striatal volume between PD patients and healthy controls.**

|  | PD | HC | p values |
| --- | --- | --- | --- |
|  | (n=130) | (n=69) |  |
| Left Caudate (cm^3^) | 1.67±0.22 | 1.65±0.22 | 0.457 |
| Right Caudate (cm^3^) | 1.73±0.26 | 1.71±0.26 | 0.507 |
| Left Putamen (cm^3^) | 2.12±0.32 | 2.13±0.34 | 0.869 |
| Right Putamen (cm^3^) | 2.03±0.28 | 2.06±0.31 | 0.476 |
| Left Striatum (cm^3^) | 5.45±0.68 | 5.45±0.70 | 0.952 |
| Right Striatum (cm^3^) | 5.47±0.66 | 5.48±0.69 | 0.855 |
| TIV (cm^3^) | 1529±164 | 1507±150 | 0.355 |

PD, Parkinson’s Disease; HC, healthy controls; TIV, Total intracranial volume.
