# Supplementary material for: Association of coffee consumption and striatal volume in patients with Parkinson's disease and healthy controls
Source: CNS Neurosci Ther. 2023 Apr 10;29(10):2800–10. doi: 10.1111/cns.14216 (PMC10493673; doi:10.1111/cns.14216)
Supplement: Supplementary file 2 — Table S2. [file CNS-29-2800-s002.docx]

**Table S2. Correlation analyses between coffee cups consumed per day and striatal volume.**

|  | PD-CC (n=80) | |  | PD-EC (n=17) | |  | HC-CC (n=47) | |  | HC-EC (n=9) | |
| --- | --- | --- | --- | --- | --- | --- | --- | --- | --- | --- | --- |
|  | Cups per day | |  | Cups per day | |  | Cups per day | |  | Cups per day | |
|  | r | p |  | r | p |  | r | p |  | r | p |
| Left Caudate | -0.074 | 0.517 |  | 0.156 | 0.579 |  | -0.248 | 0.1 |  | -0.746 | 0.054 |
| Right Caudate | -0.039 | 0.732 |  | 0.194 | 0.489 |  | -0.252 | 0.095 |  | -0.764 | 0.045 |
| Left Putamen | -0.256 | **0.023** |  | -0.075 | 0.791 |  | -0.36 | **0.015** |  | -0.643 | 0.119 |
| Right Putamen | -0.251 | **0.027** |  | -0.136 | 0.628 |  | -0.325 | **0.03** |  | -0.610 | 0.146 |
| Left Striatum | -0.221 | 0.051 |  | 0.02 | 0.945 |  | -0.376 | **0.011** |  | -0.659 | 0.107 |
| Right Striatum | -0.205 | 0.072 |  | 0.043 | 0.878 |  | -0.359 | **0.015** |  | -0.732 | 0.061 |

Bold values indicate significant differences (p < 0.05). PD-CC, current consumers of Parkinson’s Disease; PD-EC, ever consumers of Parkinson’s Disease; HC-CC, current consumers of healthy controls; HC-EC, ever consumers of healthy controls. In the correlation analyses, 80 PD-CC, 17 PD-EC, 47 HC-CC and 9 HC-EC who answered the ‘cfqa5day’ were included. ‘cfqa5day’ referred to the question “During the time you were regularly drinking caffeinated coffee, on average, about how many cups per day did you drink?”
