# Supplementary material for: Association of coffee consumption and striatal volume in patients with Parkinson's disease and healthy controls
Source: CNS Neurosci Ther. 2023 Apr 10;29(10):2800–10. doi: 10.1111/cns.14216 (PMC10493673; doi:10.1111/cns.14216)
Supplement: Supplementary file 3 — Table S3. [file CNS-29-2800-s003.docx]

**Table S3. Factors associated with volume in each striatal subregion by a multiple linear regression analysis in PD patients.**

| Striatal regions | Variables | PD (n=130) | | |
| --- | --- | --- | --- | --- |
|  |  | *ß* | 95%CI | *p* |
| Left Caudate | age | 0.014 | -0.023 to 0.024 | 0.976 |
|  | gender | 0.325 | 0.069 to 0.236 | **<0.001** |
|  | coffee consumption | 0.063 | -0.051 to 0.109 | 0.470 |
|  | smoking history | -0.027 | -0.301 to 0.222 | 0.763 |
|  | age at onset | -0.136 | -0.025 to 0.019 | 0.776 |
|  | disease duration | -0.102 | -0.009 to 0.003 | 0.270 |
|  | H-Y stages | 0.005 | -0.085 to 0.090 | 0.959 |
|  | MDS‐UPDRS III | -0.126 | -0.010 to 0.002 | 0.206 |
| Right Caudate | age | -0.913 | -0.054 to 0.002 | 0.064 |
|  | gender | 0.170 | -0.006 to 0.193 | 0.066 |
|  | coffee consumption | 0.030 | -0.078 to 0.112 | 0.730 |
|  | smoking history | -0.031 | -0.366 to 0.259 | 0.735 |
|  | age at onset | 0.710 | -0.007 to 0.046 | 0.146 |
|  | disease duration | 0.024 | -0.006 to 0.008 | 0.798 |
|  | H-Y stages | 0.109 | -0.049 to 0.160 | 0.293 |
|  | MDS‐UPDRS III | -0.160 | -0.012 to 0.001 | 0.117 |
| Left Putamen | age | -0.017 | -0.034 to 0.033 | 0.972 |
|  | gender | 0.200 | 0.011 to 0.253 | 0.033 |
|  | coffee consumption | 0.154 | -0.015 to 0.217 | 0.086 |
|  | smoking history | 0.032 | -0.313 to 0.447 | 0.726 |
|  | age at onset | -0.070 | -0.035 to 0.030 | 0.887 |
|  | disease duration | -0.053 | -0.011 to 0.006 | 0.578 |
|  | H-Y stages | 0.120 | -0.053 to 0.201 | 0.251 |
|  | MDS‐UPDRS III | -0.186 | -0.016 to 0.001 | 0.072 |
| Right Putamen | age | -0.245 | -0.037 to 0.022 | 0.611 |
|  | gender | 0.279 | 0.059 to 0.268 | **0.002** |
|  | coffee consumption | 0.110 | -0.036 to 0.164 | 0.205 |
|  | smoking history | -0.032 | -0.387 to 0.268 | 0.718 |
|  | age at onset | 0.095 | -0.025 to 0.031 | 0.843 |
|  | disease duration | -0.021 | -0.008 to 0.007 | 0.821 |
|  | H-Y stages | 0.184 | -0.009 to 0.209 | 0.073 |
|  | MDS‐UPDRS III | -0.197 | -0.015 to 0.000 | 0.050 |
| Left Striatum | age | 0.029 | -0.068 to 0.072 | 0.951 |
|  | gender | 0.300 | 0.174 to 0.677 | **0.001** |
|  | coffee consumption | 0.100 | -0.100 to 0.381 | 0.249 |
|  | smoking history | 0.032 | -0.642 to 0.935 | 0.713 |
|  | age at onset | -0.156 | -0.078 to 0.056 | 0.742 |
|  | disease duration | -0.111 | -0.029 to 0.007 | 0.230 |
|  | H-Y stages | 0.115 | -0.112 to 0.415 | 0.257 |
|  | MDS‐UPDRS III | -0.201 | -0.036 to 0.000 | 0.044 |
| Right Striatum | age | -0.573 | -0.109 to 0.026 | 0.227 |
|  | gender | 0.269 | 0.129 to 0.614 | **0.003** |
|  | coffee consumption | 0.063 | -0.145 to 0.318 | 0.461 |
|  | smoking history | -0.024 | -0.864 to 0.654 | 0.785 |
|  | age at onset | 0.360 | -0.039 to 0.089 | 0.443 |
|  | disease duration | -0.021 | -0.019 to 0.015 | 0.818 |
|  | H-Y stages | 0.202 | 0.006 to 0.514 | 0.045 |
|  | MDS‐UPDRS III | -0.222 | -0.036 to -0.002 | 0.025 |

Bold values indicate significant differences (*p* < 0.0083) after Bonferroni correction. PD, Parkinson’s Disease; HC, Healthy controls; *ß*, Standardized coefficients beta; CI: confidence interval; H‐Y stages, Hoehn & Yahr stages; MDS-UPDRS, Movement Disorders Society Unified Parkinson’s Disease Rating Scale.
